# A Ternary Composite with Medium Adsorption Confirms Good Reversibility of Li‐Se Batteries

**DOI:** 10.1002/advs.202206962

**Published:** 2023-04-14

**Authors:** Yi Li, Zhao Li, Liang Yue, Yi Zhang, Shuang Liu, Yubin Niu, Sam Zhang, Maowen Xu

**Affiliations:** ^1^ Key Lab for Advanced Materials and Clean Energies of Technologies Southwest University Chongqing 400715 P. R. China; ^2^ School of Materials and Energy Southwest University Chongqing 400715 P. R. China; ^3^ National Engineering Research Center of Light Alloy Net Forming & Shanghai Key Laboratory of Hydrogen Science School of Materials Science and Engineering Shanghai Jiao Tong University Shanghai 200240 P. R. China; ^4^ Center for Advanced Thin Films and Devices School of Materials and Energy Southwest University Chongqing 400715 P. R. China

**Keywords:** cathode–electrolyte interface films, lithium‐selenium batteries, polyselenides adsorption/desorption, reversibility

## Abstract

For Li‐Se batteries, cathode using carbonaceous hosts to accommodate Se performed modestly, whereas those applying metallic compounds with stronger chemical adsorption exhibited even more rapid capacity decay, the intrinsic reasons for which are still not clear. Herein, it is found that Se tends to precipitate on the surface of the electrode during cycling, and the precipitation speed depends on the polarization degree of the host. A further enhanced adsorption does not certainly generate better electrochemical activity, since hosts with overhigh adsorption ability are hard to desorb polyselenides, leading to catalyst passivation and rapid capacity decay. These findings encourage us to design a ternary anatase/rutile/titanium nitride (aTiO_2_/rTiO_2_/TiN@C) composite host, integrating good adsorption of TiO_2_ and rapid electron transport ability of TiN, and introducing rutile to weaken overall adsorption. The aTiO_2_/rTiO_2_/TiN@C composite with medium adsorption not only avoids rapid loss of active substances in electrolyte but also slows down the precipitation speed of Se. As a result, the aTiO_2_/rTiO_2_/TiN@C/Se electrode delivered good rate capability(154 mA h g^−1^ at 20 C) and good cycling stability(a low decay of 0.024% per cycle within 500 cycles at 2 C).

## Graphical abstract

The ternary composite not only avoids rapid dissolution of active substances in electrolyte caused by weak adsorption, but also slows down passivation of catalytic sites induced by overhigh adsorption, enabling good reversibility of Li‐Se batteries.

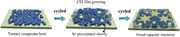

## 1 Introduction

As the representational battery system based on conversion redox reaction, lithium‐sulfur batteries undergo progressive redox reaction to transform elemental sulfur to dissolvable intermediate lithium polysulfides (LiPSs) and subsequently to final lithium sulfide.^[^ ^1^ ^]^ With a fairish potential of 2.15 V (versus Li/Li^+^) and theoretical specific capacity up to 1672 mA h g^−1^, Li‐S cells are regarded as one of promising battery candidates for next‐generation energy storage systems.^[^ ^2^ ^]^ The application of Li‐S cells is still hindered by several challenges yet, such as the severe dissolution of intermediate LiPSs, the shuttle effects between metal Li and sulfur, and the sluggish reaction dynamics.^[^ ^3^ ^]^ Many strategies have been put forward to overcome these issues, such as employing conductive carbon‐based or mental‐based host to confine active materials, and performances of cells have really been raised to some extent.^[^ ^4^ ^]^ However, the intrinsic insulation of S_8_ (≈1×10^−28^ S m^−1^) is hard to break through. In this case, looking for new cathodic materials with upper electroconductivity seems to a feasible way to get out of the dilemma.

Elemental selenium (Se) is the other one typical conversion cathode featured with a good electrical conductivity as ≈1×10^−3^ S m^−1^ and a high energy density of 2528 Wh L^−1^ with a theoretical specific capacity of 675 mA h g^−1^.^[^ ^5^ ^]^ The as‐constructed Li‐Se batteries undergoes similar sequential phase transition as Li‐S batteries that original substances turn to lithium polyselenides (LiPSes) and next to lithium selenide (Li_2_Se).^[^ ^6^ ^]^ Differently, in comparison to ether‐based electrolyte employed by Li‐S batteries, the most frequently used carbonate‐based electrolyte utilized by Li‐Se batteries demonstrates a lower cost but a better cell capacity retention thanks to where less solubilization of LiPSes occurs and minor nucleophilic reaction of Se with carboxide takes place.^[^ ^7^ ^]^ Whereas, the hard truth is that the dissolution of LiPSes and the shuttle effect and slack kinetics resulting from multielectron and multiphase electrochemical reactions are still the major factor affecting cell performance.^[^ ^8^ ^]^

Consulting strategies of Li‐S batteries, researchers utilized suitable host materials to accommodate Se. Carbon materials, such as porous carbon, carbon nanotubes, and graphene, are the most commonly used host materials, which provide space to buffer volume changes during cycling, and provide certain physical adsorption to inhibit the diffusion of LiPSes.^[^ ^9a–c^ ^]^ A small number of pioneers applied transition metals/metallic compounds as host of Se. Unlike carbon materials with physical attraction, metals/metallic compounds have better adsorption toward soluble LiPSes through forming chemical bonds, preventing LiPSes from unbounded dissolution in electrolyte.^[^ ^10a,b^ ^]^ More importantly, the chemical adsorption creates forceful pull on Se_8_/LiPSes molecules and ultimately makes Se—Se bonds rupture easily, namely to lower potential barrier for chemical reaction to occur. Referring to previous researches on Li‐S batteries, metallic compounds such as metal oxides, sulfides, and selenides could benefit whether S or Se by adsorb soluble intermediates and catalyze the conversion reaction.^[^ ^11^ ^]^ And when researchers screen catalyst, a seemed recognized principle is that the stronger adsorption is the less dissolution of active substance is, and the better the cell performance is. For example, metal oxides endow plenty of polar sites to anchor adsorb soluble intermediates and relieve shuttle effect.^[^ ^12a,b^ ^]^ Moreover, to further ease dissolution, approaches were proposed to improve the adsorption capability of metal oxides, such as changing the band structure of metal oxides and designing a metal oxides/nitrides (or sulfides, carbides) heterostructure to enhance binding energy with LiPSs.^[^ ^13a,b^ ^]^ Since selenium has similar properties with sulfur, strategies of Li‐S batteries seem feasible for reference. However, few studies have been reported and the existed researches performed not as well as expected, and the reason is still not clear.

On the other hand, for electrocatalysis, volcano‐shaped figure reflects the relation of electrocatalytic activity with bond energy, as only suitable binding energy exhibits high electrocatalytic activity.^[^ ^14a–c^ ^]^ If binding energy is too low, increasing adsorption is required to improve catalytic activity. Whereas if binding energy is too high, the catalysts cannot be easily desorbed after adsorption. Then catalysts gradually passivated, and the reversibility further deteriorated. Therefore, taking the reversibility into consideration, how to improve the catalytic activity for Li‐Se cells is worthy of discussion.

Contrary to common viewpoint of improving adsorption of host materials, in this article we proposed a strategy that weakening adsorption ability of polar materials to ensure better capacity retention for Li‐Se batteries. A series of Ti‐MOF derived aTiO_2_@C, aTiO_2_/rTiO_2_/TiN@C, TiN@C, and nitrogen‐doped porous carbon (NC) were synthesized as host of Se. By examining electrochemical activity to LiPSes and cell performance of as‐assembled Li‐Se batteries, we found the stronger adsorption capacity does not certainly result in better cell performance. Ti‐based materials are supposed to be difficult to desorb after rapidly adsorbing, which gradually evolved as catalyst passivation, poor battery reversibility, and rapid capacity decay. Therefore, rTiO_2_ was introduced to weaken the overall adsorption capability. The designed aTiO_2_/rTiO_2_/TiN@C composite, not only avoided rapid loss of active substances in electrolyte but also slowed down the irreversible precipitation of Se on the surface of electrode. As a result, the ineluctable precipitation of Se on the surface of electrode was coated and reserved by slow‐growing cathode–electrolyte interface (CEI) film, and the aTiO_2_/rTiO_2_/TiN@C/Se electrode delivered good rate capability and good cycling stability. This study may offer a novel insight on catalyst design of Li‐Se batteries in the future.

## 2 Results and Discussion

The visible adsorption experiment was done first to rank the adsorption capability of pure commercial aTiO_2_, rTiO_2_, and TiN. Under the same conditions, solution color with aTiO_2_ and TiN faded obviously while that of rTiO_2_ hardly changes (Figure S1a, Supporting Information). Upper solution was taken to do UV–vis test, the result also confirmed stronger adsorption ability of aTiO_2_ and TiN than rTiO_2_ (Figure S1b, Supporting Information). Usually, researchers tend to acquire both strong adsorption and rapid electron transportation by integrate aTiO_2_ and TiN.^[^ ^15^ ^]^ Here the ternary composite introduced with rutile is supposed to weaken adsorption and maintain good electron transportation.

**Figure 1:**
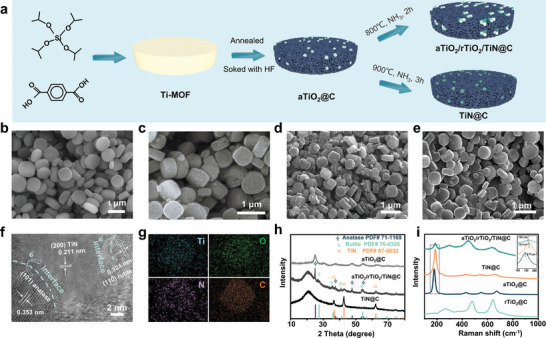
Morphology and Structural characterizations. a) Schematical diagram of preparation procedure of host materials, b–e) SEM images of Ti‐MOF precursor, aTiO_2_@C, aTiO_2_/rTiO_2_/TiN@C, and TiN@C host, f,g) TEM images and element mapping images of aTiO_2_/rTiO_2_/TiN@C, h) XRD pattern, i) Raman spectra of host materials.

The preparation route of host materials could be described as **Figure** **1**a. Ti‐MOF shows a fine crystallinity and a uniform size distribution (Figure 1b and Figure S2, Supporting Information). After been annealed at the temperature of 600 °C under Ar and soaked in HF solution to remove redundant TiO_2_, the precursors turned into aTiO_2_@C. The surface became unsmooth and the size dwindled (Figure 1c). Next, aTiO_2_@C powder was annealed under NH_3_ atmosphere to produce aTiO_2_/rTiO_2_/TiN@C, while NC was obtained under the same annealing procedure after remove TiO_2_ completely. TiN@C was reaped at a lengthen annealing time and upper temperature. This unique ternary aTiO_2_/rTiO_2_/TiN@C composite, featured with a rough morphology with particles distributed on the surface or embedded inside of materials, showed no shrink in size anymore (Figure 1d). The TEM images verified pore‐rich and particle‐rich traits of the aTiO_2_/rTiO_2_/TiN@C composite (Figure S3a,b, Supporting Information). The high‐resolution TEM image confirms three components of material, as illustrated in Figure 1f, and the lattice‐fringe distance of 0.324 nm on the upper‐right corner belongs to (1 1 0) plane of rutile, as for another lattice‐fringe distances of 0.353 and 0.211 nm are corresponding to (1 0 1) and (2 0 0) planes of anatase and TiN respectively, which are evidences for successful construction of ternary composite.^[^ ^16^ ^]^ Notably, the interfaces between two phase could be easily found, indicating heterostructure of aTiO_2_/rTiO_2_/TiN@C, which further do benefits for rapid electrons migration on cycling. In EDS element mapping of aTiO_2_/rTiO_2_/TiN@C composite, Ti, N, O, and C element are well‐distributed (Figure 1g). XRD patterns of these host materials were listed at Figure 1h, which are highly in accordance with PDF cards. For aTiO_2_/rTiO_2_/TiN@C composite, the sharp diffraction peaks are in line with anatase (PDF#71‐1169), rutile (PDF#75‐1757), and TiN (PDF#87‐0632), which testified three types of phases.^[^ ^17^ ^]^ And the mass ratio of three substance in the ternary composite was revealed by XRD refinement as aTiO_2_:rTiO_2_:TiN ≈ 43.7:39.6:16.7 (Figure S4, Supporting Information). The content of carbon in host materials were controlled almost unanimous as 63.8 wt% in aTiO_2_@C, 64% wt% in TiN@C, and 63.9% in aTiO_2_/rTiO_2_/TiN@C (Figure S5, Supporting Information). Raman spectra was carried out as Figure 1i. When carefully analyze the structure of N—Ti—O polar bond, one can easily suppose that a longer bond length of Ti—N bond but a shorter bond length of Ti—O bond than in normal TiN and TiO_2_, since the higher electronegativity of oxygen atom than that of nitrogen atom will make electron cloud of titanium lean to the side of oxygen atom, which further expresses as red shift (toward low Raman shift) of Ti—N bond and blue shift (toward high Raman shift) of Ti—O bond in Raman patterns. On the Raman pattern of aTiO_2_/rTiO_2_/TiN@C, the band at 185 cm^−1^ resulted from slightly red shift of Ti—N at 187 cm^−1^ and slightly blue shift of Ti—O (aTiO_2_) at 177 cm^−1^, while the band at 294 cm^−1^ could be deemed as the result of blue shift of Ti—O (rTiO_2_) at 271 cm^−1^.^[^ ^16^ ^]^ Raman patterns are favorable evidences for existence of N—Ti—O polar bonds, which testified the heterostructure. In XPS patterns, Ti 2p spectrum (Figure S6a, Supporting Information) was split to three couples of peaks, corresponding to Ti—O, Ti—N, O—Ti—N bonds respectively.^[^ ^18^ ^]^ N 1s spectrum (Figure S6b, Supporting Information) was separated to three peaks, representing N—Ti, O—N—Ti, graphite N respectively, and O 1s spectrum (Figure S6c, Supporting Information) could be divided into three peaks, as O—Ti, O—N, O—H bonds respectively.^[^ ^19^ ^]^ These XPS datum are proofs for successful construction of hybrid composite. Additionally, some of N atoms were doped in carbon matrix (bonded with C) and further enhanced electroconductibility of materials (Figure S6d, Supporting Information).

After being loaded with Se, the morphology of matrixes remained almost unchanged, indicating structural stability, as shown in Figure S7a–d, Supporting Information. The EDS liner scanning result of aTiO_2_/rTiO_2_/TiN@C/Se showed Ti, N, O, Se, C elemental distribution on single particle, it is seen that Se takes the maximum content (Figure S8a,b, Supporting Information). While in XRD patterns of aTiO_2_@C/Se, aTiO_2_/rTiO_2_/TiN@C/Se, TiN@C/Se, and NC/Se, no characteristic peaks of Se are observed, revealing amorphous state of Se in these composites (Figure S9, Supporting Information). And only the main peak of host materials could be barely detected, as result of low contents in the Se‐based composite and coverage of molten Se hindered detection of X‐ray.^[^ ^20^ ^]^ The mass percentage of Se in composites were figured up as high as 58 wt% in aTiO_2_@C/Se, 59.5 wt% in aTiO_2_/rTiO_2_/TiN@C/Se, 60 wt% in NC/Se, and 61 wt% in TiN@C/Se by a TGA measurement under a nitrogen flow (Figure S10, Supporting Information).

**Figure 2:**
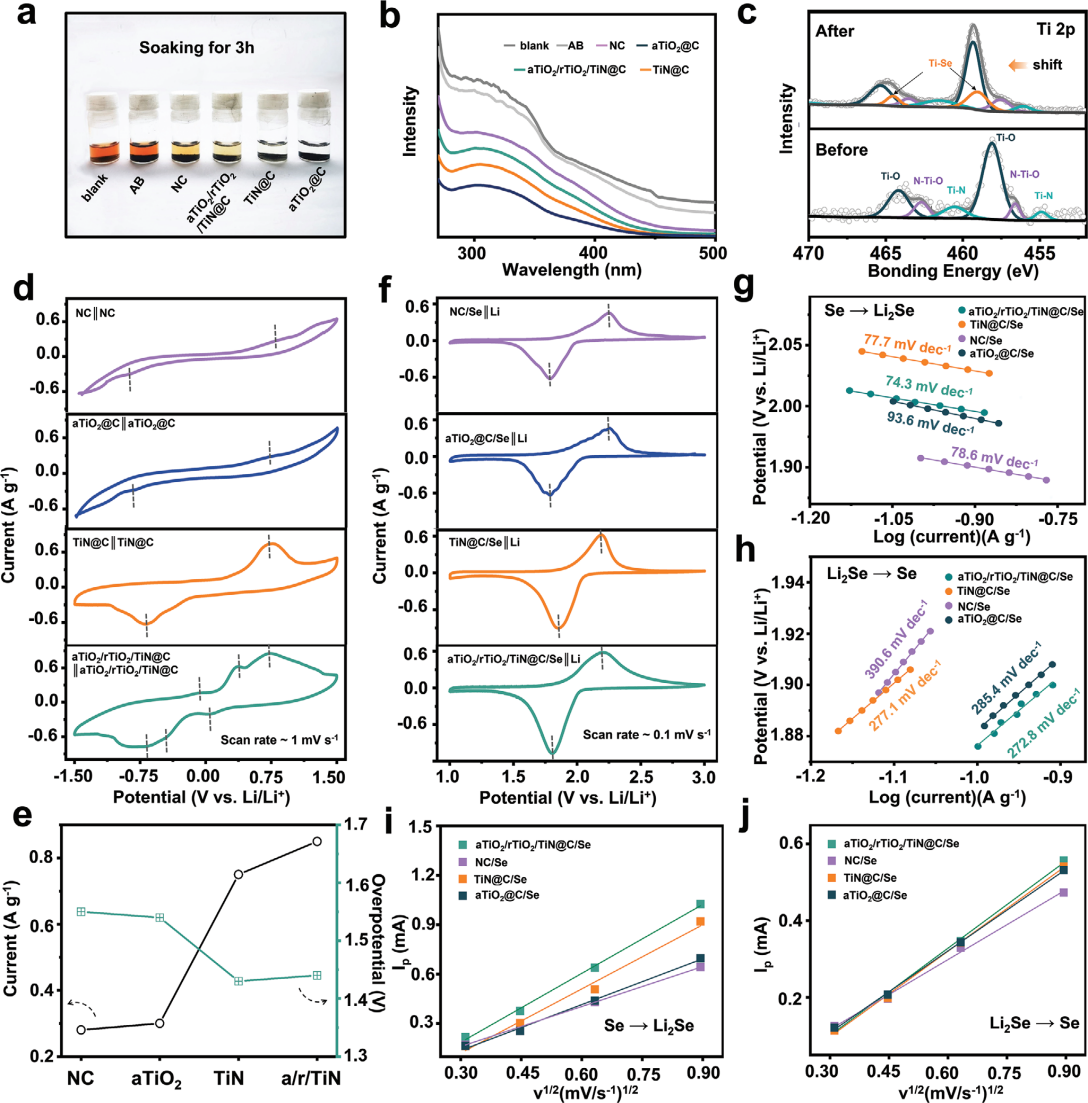
Adsorption and catalysis activity characterizations. a) digital photos of LiPSes adsorption experiment, b) UV–vis spectrum with host materials after immersed in Li_2_Se*_n_* solution for 3 h, c) Ti 2p XPS spectra (aTiO_2_/rTiO_2_/TiN@C) before and after adsorption, d) CV plots of symmetric cells with different electrodes, e) peak current and voltage gaps between anodic and cathodic CV peaks f) CV curves, g,h) Tafel plots for the conversion reactions, i,j) the liner relation between *v* ^1/2^ and *I*_p_.

To confirm the weakened adsorption ability of as‐synthesized ternary aTiO_2_/rTiO_2_/TiN@C composite toward soluble LiPSes, the visible adsorption experiment was launched. After 3 h, the solution immersed aTiO_2_@C and TiN@C became almost colorless, while the color of solution immersed aTiO_2_/rTiO_2_/TiN@C and NC faded obviously and the solution immersed AB showed almost no change compared with blank Li_2_Se*_n_* solution (**Figure** **2**a). All the supernatant was acquired and examined by a UV–vis spectrophotometer, and the result showed that the intensity for Li_2_Se*_n_* solution was of the decreasing order among AB, NC, aTiO_2_/rTiO_2_/TiN@C, TiN@C, aTiO_2_@C (Figure 2b). The result indicated that both aTiO_2_@C and TiN@C have strong adsorption ability toward Li_2_Se*_n_*, while aTiO_2_/rTiO_2_/TiN@C has a medium adsorption. Additionally, aTiO_2_/rTiO_2_/TiN@C powders after adsorption were taken to observe changes of chemical state. Compared with the original survey spectra, in the survey spectra after adsorption emerged signals of Se (Figure S11a,b, Supporting Information).^[^ ^21^ ^]^ All the peaks of Ti shift to higher bonding energy, which testified Ti as electrons donor to Li_2_Se*_n_*, and the newly formed Ti—Se bonds are convincing evidence of chemical interaction between Li_2_Se*_n_* and Ti (Figure 2c).^[^ ^22^ ^]^ Cyclic voltammetric tests of symmetrical cells were conducted to evaluate LiPSes conversion capacity of these host materials (Figure 2d), and the result showed that NC and aTiO_2_@C symmetrical cell exhibited lower current signal. TiN@C symmetrical cell showed a couple of symmetrical redox peaks while the aTiO_2_/rTiO_2_/TiN@C symmetrical cell displayed larger peak area and three couples of redox peaks, implying good catalysis of TiN@C and even better catalysis of aTiO_2_/rTiO_2_/TiN@C toward LiPSes.^[^ ^23^ ^]^ The aTiO_2_/rTiO_2_/TiN@C symmetrical cell possessed the maximal current area among four materials (Figure 2e), indicating the highest utilization of Se. In cathodic section of CV curves of the as‐made Se‐based electrodes, the TiN@C/Se electrode exhibited upper onset potential and peak voltage while aTiO_2_/rTiO_2_/TiN@C/Se electrode demonstrated larger peak area than other counterparts (Figure 2f). Contrastively, in anodic section of CV curves, aTiO_2_/rTiO_2_/TiN@C/Se exhibited lower onset potential and equal peak voltage with TiN@C/Se electrode. Additionally, the calculated Tafel slopes of redox peaks revealed that aTiO_2_/rTiO_2_/TiN@C/Se electrode had lower slope values as 74.3/278.2 mV dec^−1^ in anodic/cathodic sweep compared with NC/Se, aTiO_2_@C/Se, and TiN@C/Se electrode (Figure 2g,h), implying its superior redox kinetics during charge/discharge and good electrocatalysis toward phase transition.^[^ ^24^ ^]^ The lithium‐ion diffusion rates (D_Li_^+^) of as‐assembled cells were obtained by means of studying CV curves at different scan rates (Figure S12a–d, Supporting Information). According to the Randles−Sevcik equation, the peak current (*I*_p_) was linearly dependent on the square root of the scan rate (*ν* ^1/2^), and the D_Li_^+^ was positively related to the ratio of *I*_p_/*ν* ^1/2^.^[^ ^25^ ^]^ In anodic sweep (corresponding to Se transformed into Li_2_Se), the slope value of TiO_2_/rTiO_2_/TiN@C/Se and TiN@C/Se were higher than that of aTiO_2_@C/Se and NC/Se, suggesting a faster Li‐ions migration rate (Figure 2i). Similarly, in cathodic sweep (corresponding to Li_2_Se converted into Se), the slope value of TiO_2_/rTiO_2_/TiN@C/Se and TiN@C/Se were higher than other two electrodes, implying good capability of Li‐ions migration in both charge/discharge process (Figure 2j).

**Figure 3:**
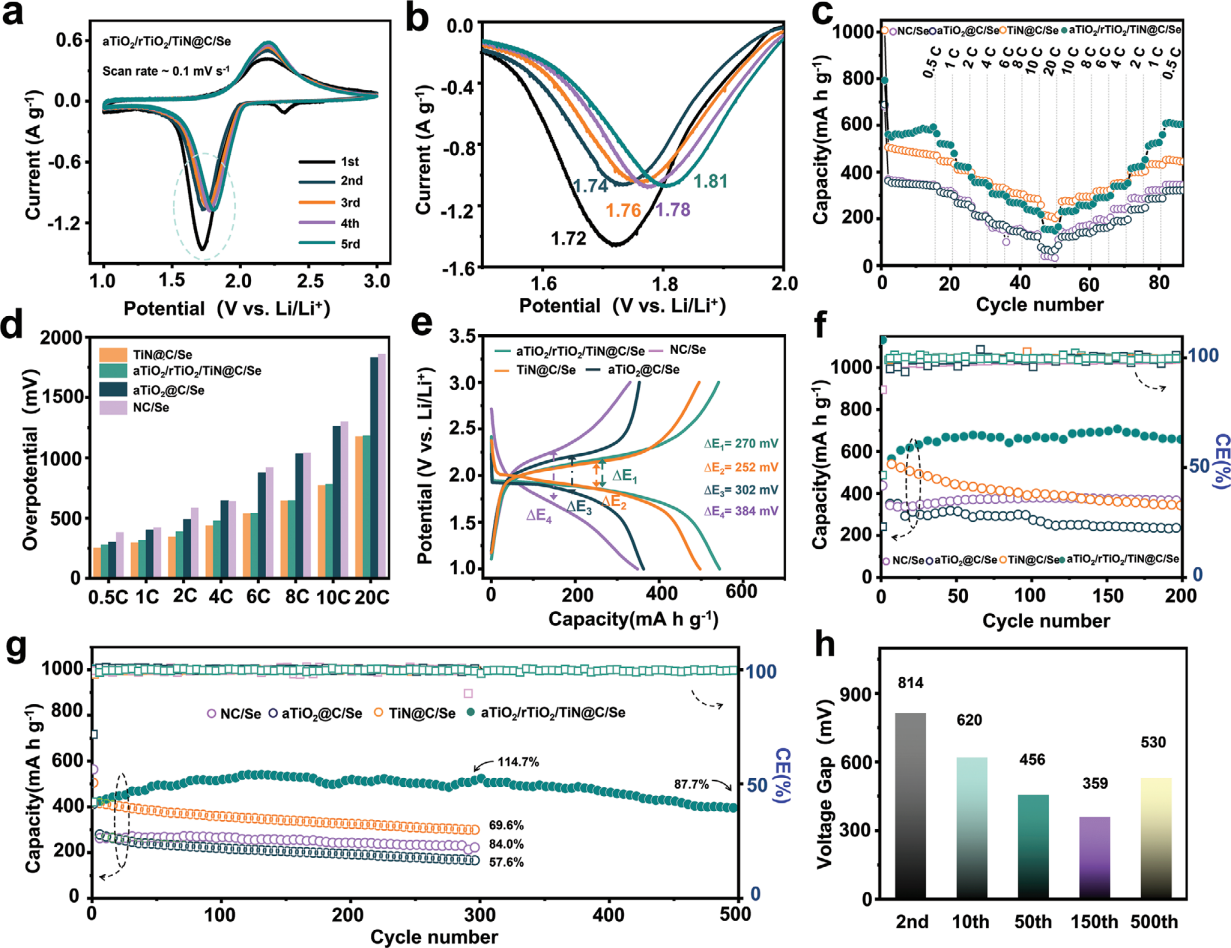
Electrochemical performance. The electrochemical performance of Li‐Se batteries a,b) CV curves of aTiO_2_/rTiO_2_/TiN@C/Se electrode at a scan rate of 0.1 mV s^−1^, c) rate performances, d) charge–discharge overpotentials at different C‐rate, e) charge–discharge profiles at 0.5 C, cyclic performance at f) 0.5 C, g) 2 C, h) charge–discharge overpotentials of aTiO_2_/rTiO_2_/TiN@C/Se electrode at different cycles.

The Li‐Se batteries were assembled by coupling as‐synthesized cathode with Li‐metal anode under a regular electrolyte/Se ratio of 25 µL mg^−1^ to examine electrochemical performance (based on ester electrolyte).^[^ ^26^ ^]^ In the CV curves of the first cycle, the peak at 2.33 V is related to formation of CEI originating from nucleophilic reaction between LiPSes and electrolyte (**Figure** **3**a).^[^ ^27^ ^]^ The redox peaks, located at 1.72 V/2.20 V corresponding to formation of Li_2_Se and regeneration of Se respectively, shifted to 1.81 V/2.19 V after 5 cycles, indicating polarization of electrochemical reaction was diminishing gradually (Figure 3b).^[^ ^28^ ^]^ The EIS curves of the first, tenth, 20th, 30th, and 50th cycle illustrated two semicircles, representing the double‐layer and CEI layer respectively (Figure S13, Supporting Information). The small radius and minor variation of semicircles during cycling indicate low electron transfer impedance and stable CEI layer, implying good rate capability and good cycle performance, and the slopes of plots in high frequency region grew along cycling times, indicating faster mass diffusion dynamic during cycling.^[^ ^7^ ^]^ Rate performance of aTiO_2_/rTiO_2_/TiN@C/Se electrode was evaluated at current densities from 0.5 to 20 C. As shown in Figure 3c, the cell delivered discharge capacities as high as 550, 519, 424, 356, 305, 258, 233, 154 mA h g^−1^ at current densities of 0.5, 1, 2, 4, 6, 8, 10, 20 C. When the current density was back to 0.5 C, the cell displayed increased capacities of 235, 257, 291, 356, 424, 524, 605 mA h g^−1^, implying good capability of rapid charge/discharge. In contrast, the NC/Se electrode showed lower capacities at the identical rates, and aTiO_2_@C/Se electrode delivered almost as much capacities as NC/Se electrode but lower capacity when current density reversed back, which infers worse reversibility of aTiO_2_@C/Se electrode. The TiN@C/Se electrode delivered 1008 mA h g^−1^ at 0.5 C in the beginning and faded to 472 mA h g^−1^, and discharged a capacity of 201 mA h g^−1^ at 20 C. When the current density was back to 0.5 C, the cell cannot deliver same capacities as before, intimating TiN@C/Se electrode had worse reversibility. The overpotential of different rate indicated that aTiO_2_/rTiO_2_/TiN@C/Se and TiN@C/Se electrodes showed lower polarization than the other two (Figure 3d). Figure 3e demonstrated aTiO_2_/rTiO_2_/TiN@C/Se owned similar overpotential as TiN@C/Se at 0.5 C, and both of them were lower than aTiO_2_@C/Se and NC/Se electrode. The cycling performance of as‐prepared electrodes at a current density of 0.5 C were tested (Figure 3f). The TiN@C/Se and aTiO_2_@C/Se electrode showed good capacity at the beginning and fell off rapidly, and the NC/Se exhibited a slight increase in capacity and then attenuated. The aTiO_2_/rTiO_2_/TiN@C/Se electrode displayed a discharge capacity of 1131 mA h g^−1^ over theoretical specific capacity (675 mA h g^−1^) at the first cycle, which were supposed to stem from the inevitably irreversible reaction of electrolyte with electrode materials and dissolution of some LiPSes molecules.^[^ ^29^ ^]^ Then the discharge capacity dropped to ≈590 mA h g^−1^ then gradually increased to 700 mA h g^−1^ at the 150th cycle and finally faded to 657 mA h g^−1^ at the 200th cycle. The little capacity contribution of the bare AB/Se and aTiO_2_/rTiO_2_/TiN@C electrode proved that the above good cyclic performance came from reasonable construction of aTiO_2_/rTiO_2_/TiN@C/Se composite (Figures S14a,b and S15, Supporting Information). This capacity growth‐fading phenomenon still existed in cycling tests at 2 C rate (Figure 3g). The aTiO_2_/rTiO_2_/TiN@C/Se electrode delivered 449 mA h g^−1^ at the second cycle, and then grew to 532 mA h g^−1^ at the 150th cycle, and gradually faded to 515 mA h g^−1^ (114.7% of capacity retention from the second cycle) at 300th cycle, and finally faded to 394 mA h g^−1^ (87.7% of capacity retention from the second cycle) at the 500th cycle (attenuation rate ≈0.024% per cycle, based on the second cycle). As comparison, the NC/Se electrode had a slight capacity rise at the beginning and then faded, and the aTiO_2_@C/Se and TiN@C/Se electrode continued to decline. The capacity retention of NC/Se, aTiO_2_@C/Se and TiN@C/Se cycled for 300 times was 84.0%, 57.6%, and 69.6% respectively. Concerning with the adsorption ability toward LiPSes, it is supposed that the weakened adsorption ability of aTiO_2_/rTiO_2_/TiN@C/Se would lead to better capacity retention than that is strong. Besides, the corresponding capacity‐potential curves (Figure S16, Supporting Information) of aTiO_2_/rTiO_2_/TiN@C/Se at the second, tenth, 50th, 150th, 500th cycle exhibited voltage gap of 814, 620, 456, 359, 530mV respectively, revealing the first decrease and then increase of electrode polarization (Figure 3h). Even when the electrolyte was reduced to 8 µL mg^− 1^ and 10 µL mg^−1^ (Figure S17, Supporting Information), the maximum values of capacity appear in advance and lie at ≈70th cycle and ≈40th cycle respectively, indicating the capacity grow‐fading phenomenon are closely related to electrolyte. Finally, the cells were disassembled after cycled for 20 times to observe morphology of Li anode (Figure S18, Supporting Information). By contrast, Li‐metal coupled with aTiO_2_/rTiO_2_/TiN@C/Se electrode demonstrated the slightest corrosion, indicating the aTiO_2_/rTiO_2_/TiN@C host avoids dissolution/diffusion of Se species effectively.^[^ ^30^ ^]^

**Figure 4:**
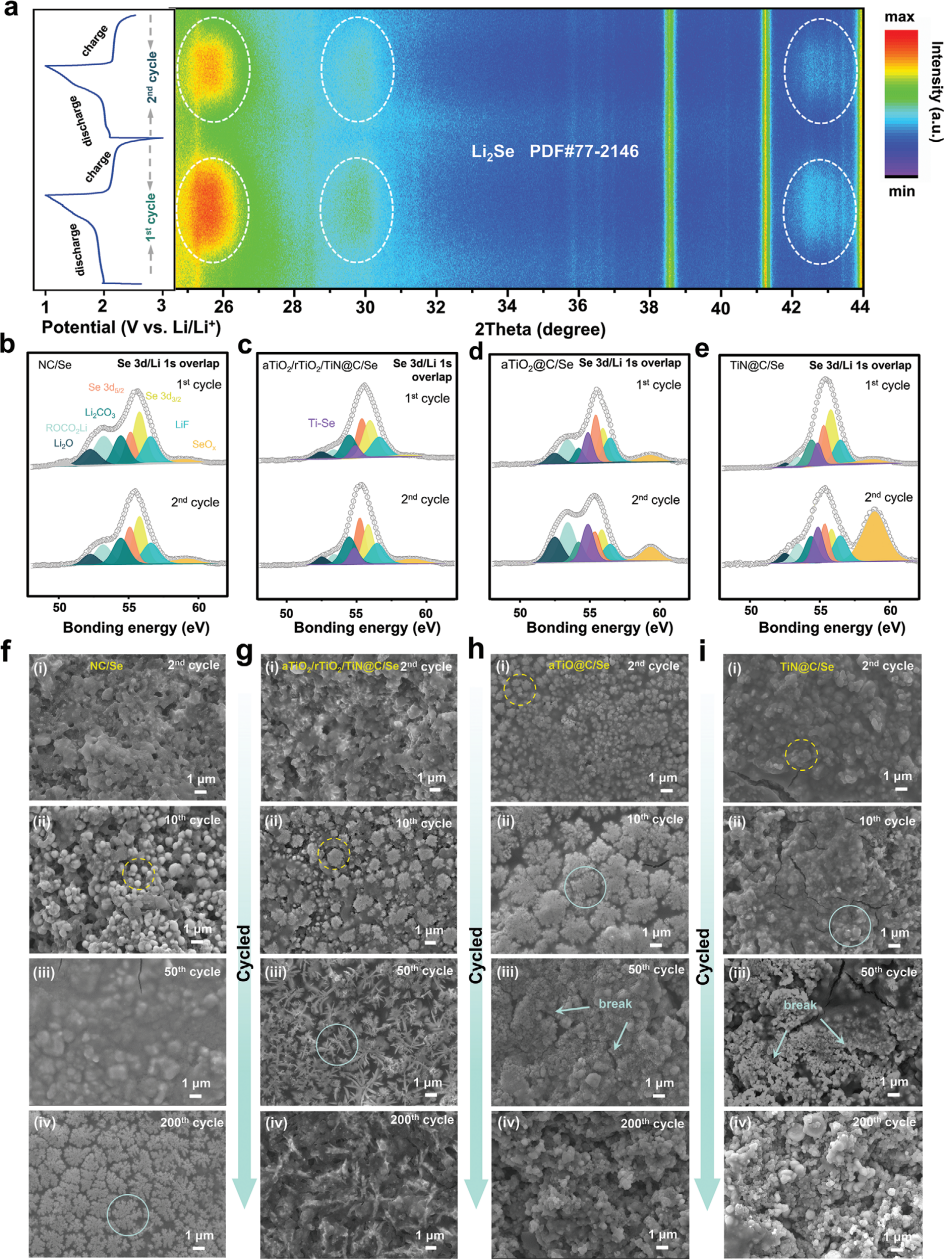
Chemical state and morphology change characterizations. a) In situ XRD of Li‐Se batteries based on aTiO_2_/rTiO_2_/TiN@C/Se electrode, ex situ Se 3d and Li 1s XPS spectra of b) NC/Se, c) aTiO_2_/rTiO_2_/TiN@C/Se, d)aTiO_2_@C/Se, e)TiN@C/Se electrode after first and second cycle, the SEM images of electrode f) NC/Se, g) aTiO_2_/rTiO_2_/TiN@C/Se, h) aTiO_2_@C/Se, i) TiN@C/Se after second, tenth, 50th, and 200th cycle respectively.

To figure out origins of the unusual capacity grow‐fading phenomenon of aTiO_2_/rTiO_2_/TiN@C/Se electrode, in situ XRD detection during the first two cycles at a current of 0.5 C was measured (**Figure** **4**a). On account of the amorphous state of Se accommodated in the host, none of characteristic peak of Se was observed on the beginning. In the first cycle, three characteristic peaks (at 25.7°, 29.7°, 42.5°) of Li_2_Se emerged when the cell was discharged to 1.9 V, and existed until the cell was charged to 2.2 V, which was well in line with electrochemical redox reaction of batteries.^[^ ^31^ ^]^ Whereas in the second cycle, the peaks of Li_2_Se appeared at an advance voltage of 1.95 V, whose intensity decreased obviously compared to the first cycle. The origin has been explained before.^[^ ^27^ ^]^ Furthermore, XPS technology was utilized to check the chemical states of Se after first, second cycles. After cycled for once, the CEI film composed of Li_2_O, ROCO_2_Li, Li_2_CO_3_, and LiF was generated on the surface of all of electrodes, which was supposed to be the reason of continued growth in capacity.^[^ ^32^ ^]^ And for NC@Se electrode (Figure 4b), except for a little increasing of SeO*_x_* peak, the spectrum of Se 3d and Li 1s demonstrated hardly any change at the second cycle compared to the first, indicating good reversibility. The aTiO_2_/rTiO_2_/TiN@C/Se electrode showed good reversibility as well, in spite of a slight enhancement of Ti—Se and SeO*_x_* peaks (Figure 4c).^[^ ^20^ ^]^ Contrastively, aTiO_2_@C/Se and TiN@C/Se electrodes displayed significant change in peak pattern, and especially the intensity of SeO*_x_* peaks increased sharply (Figure 4d,e), exhibiting poor reversibility. It is notable that Ti—Se bond in aTiO_2_@C/Se and TiN@C/Se electrodes after first cycle were higher than in aTiO_2_/rTiO_2_/TiN@C/Se electrode, and its intensity grew even stronger after cycled for the second time. This result indicated that active materials adsorbed by pure aTiO_2_ or TiN were supposed to be difficult to desorb during cycling, which is the origin of rapid capacity decay of aTiO_2_@C/Se and TiN@C/Se electrodes, while the ternary aTiO_2_/rTiO_2_/TiN@C host retarded this irreversible issue. Additionally, the morphology of electrodes after cycled for 2, 10, 50, 200 times was observed by SEM (Figure 4f‐i). For all the electrodes, compared to the original electrode (Figure S19, Supporting Information), the CEI film grown on the surface of electrode could be observed from the second cycle, looked like a dense gel and composited of complex elements (Figure S20, Supporting Information). Then CEI film gradually grow into a whole piece along with selenium precipitated out (Figure S21, Supporting Information). During cycling, LiPSes tends to gradually nucleate on the surface of electrode where the catalyst locates (for maximum polarity), as Figure 4f‐ii (tenth cycle), Figure 4g‐ii (tenth cycle), Figure 4h‐i (second cycle), and Figure 4i‐i (second cycle) marked. As circulation continued, Se went to being well exposed on the surface of electrodes, as shown in Figure 4f‐iv (200th cycle), Figure 4g‐iii (50th cycle), Figure 4h‐ii (tenth cycle), and Figure 4i‐ii (tenth cycle). And finally, after repeated charge/discharge, the CEI film was broken under sustained stress (Figure S22, Supporting Information, Figure 4h‐iii,i‐iii). The SEM detection demonstrated that for Se precipitation speed and CEI film broken speed, aTiO_2_@C/Se and TiN@C/Se electrodes are more rapid than NC and aTiO_2_/rTiO_2_/TiN@C/Se.

**Figure 5:**
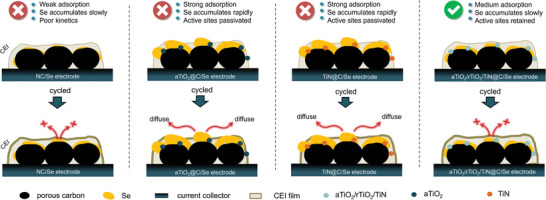
Schematic diagram of the electrode variation during cycling.

The origin of difference in performance among electrodes could be elucidated as **Figure** **5**. First, the growth of CEI film provided capacity contribution and polarization decrease during cycling. Second, the precipitation of Se brought about loss of active substance and capacity attenuation. Third, the stronger adsorption made the faster precipitation. NC/Se electrode was weak in adsorption, then Se precipitated slowly and well‐protected by the slow‐growing CEI film, thus it had the best reversibility, and even obtained a slight capacity increasement during cycling. But lack of catalyst, the cell was poor in kinetics. For aTiO_2_@C/Se and TiN@C/Se electrodes, the strong adsorption made active substance precipitated rapidly and desorbed difficultly, which covered on catalyst sites and evolved as catalyst passivation. The slow‐growing CEI film had no time to protect active substance precipitated out, then active substance dissolution and rapid capacity decay occurred. Comparatively, the ternary aTiO_2_/rTiO_2_/TiN@C/Se electrode has medium adsorption, thus active substance precipitated in a moderate speed. The slow‐growing CEI film had enough time to coat which precipitated out, then a good capacity retention with good redox kinetics were harvest. Namely, capacity growth‐fading phenomenon was related to the growing of CEI film in initial stage and broken in later stage, as protection and loss of protection to active substance. As a result, the aTiO_2_/rTiO_2_/TiN@C/Se electrode delivered the highest capacity even compared to other host materials made in this work and relevant published works (Figure S23, Supporting Information).^[^ ^5^, ^9^, ^10^, ^21^, ^33^, ^34^, ^35^ ^]^

## 3 Conclusions

The medium adsorption of ternary aTiO_2_/rTiO_2_/TiN@C composite not only avoids rapid loss of active substances in electrolyte but also slows down the irreversible precipitation of Se on the surface of electrode, helping the as‐constructed electrode acquire good reversibility. The Se precipitated on the surface of electrode was coated and reserved by slow‐growing CEI film, and the aTiO_2_/rTiO_2_/TiN@C/Se electrode delivered good rate capability as 154 mA h g^−1^ at 20 C and good cycling stability as a low decay of 0.024% per cycle within 500 cycles at 2 C rate.

## Conflict of Interest

The authors declare no conflict of interest.

## Supporting information

Supporting Information

## Data Availability

The data that support the findings of this study are available from the corresponding author upon reasonable request.

## References

[1] A. Manthiram , Y. Fu , S.‐H. Chung , C. Zu , Y.‐S. Su , Chem. Rev. 2014, 114, 11751. 25026475 10.1021/cr500062v

[2] X. Ji , K. T. Lee , L. F. Nazar , Nat. Mater. 2009, 8, 500. 19448613 10.1038/nmat2460

[3] Y. Yang , M. T. McDowell , A. Jackson , J. J. Cha , S. S. Hong , Y. Cui , Nano Lett. 2010, 10, 1486. 20184382 10.1021/nl100504q

[4] Y. Chen , T. Wang , H. Tian , D. Su , Q. Zhang , G. Wang , Adv. Mater. 2021, 33, 2003666. 10.1002/adma.202003666 34096100

[5] Z. Li , L. Yuan , Z. Yi , Y. Liu , Y. Huang , Nano Energy 2014, 9, 229.

[6] X. Li , J. Liang , J. T. Kim , J. Fu , H. Duan , N. Chen , R. Li , S. Zhao , J. Wang , H. Huang , X. Sun , Adv. Mater. 2022, 34, 2200856. 10.1002/adma.202200856 35365923

[7] X. Gu , T. Tang , X. Liu , Y. Hou , J. Mater. Chem. A 2019, 7, 11566.

[8] Y. Dong , P. Lu , Y. Ding , H. Shi , X. Feng , Z.‐S. Wu , SusMat 2021, 1, 393.

[9] a) C.‐P. Yang , Y.‐X. Yin , Y.‐G. Guo , J. Phys. Chem. Lett. 2015, 6, 256; 26263460 10.1021/jz502405h

[10] a) H. Tian , H. Tian , S. Wang , S. Chen , F. Zhang , L. Song , H. Liu , J. Liu , G. Wang , Nat. Commun. 2020, 11, 5025; 33024100 10.1038/s41467-020-18820-y PMC7538427

[11] J. He , W. Lv , Y. Chen , J. Xiong , K. Wen , C. Xu , W. Zhang , Y. Li , W. Qin , W. He , J. Power Sources 2017, 363, 103.

[12] a) Z. Zhang , X. Yang , X. Wang , Q. Li , Z. Zhang , Solid State Ion 2014, 260, 101;

[13] a) Q. Hao , G. Cui , Y. Zhang , J. Li , Z. Zhang , Chem. Eng. J. 2020, 381, 122672;

[14] a) M. D. Wodrich , B. Sawatlon , M. Busch , C. Corminboeuf , Acc. Chem. Res. 2021, 54, 1107; 33570407 10.1021/acs.accounts.0c00857

[15] J. Cai , Z. Sun , W. Cai , N. Wei , Y. Fan , Z. Liu , Q. Zhang , J. Sun , Adv. Funct. Mater. 2021, 31, 2100586.

[16] L. Ma , L.‐J. Yu , J. Liu , Y.‐Q. Su , S. Li , X. Zang , T. Meng , S. Zhang , J. Song , J. Wang , X. Zhao , Z. Cui , N. Wang , Y. Zhao , Energy Storage Mater. 2022, 44, 180.

[17] N. Li , L. Yu , J. Xi , Small 2021, 17, e2103001. 34331399 10.1002/smll.202103001

[18] C. Qi , M. Cai , Z. Li , J. Jin , B. V. R. Chowdari , C. Chen , Z. Wen , Chem. Eng. J. 2020, 399, 125674.

[19] J. Li , Y. Li , Q. Lan , Z. Yang , X.‐J. Lv , J. Power Sources 2019, 423, 166.

[20] X. Zhao , L. Yin , T. Zhang , M. Zhang , Z. Fang , C. Wang , Y. Wei , G. Chen , D. Zhang , Z. Sun , F. Li , Nano Energy 2018, 49, 137.

[21] P. Xue , Y. Zhai , N. Wang , Y. Zhang , Z. Lu , Y. Liu , Z. Bai , B. Han , G. Zou , S. Dou , Chem. Eng. J. 2020, 392, 123676.

[22] P. Behera , S. Bera , M. M. Patidar , A. K. Mishra , M. Krishnan , R. Venkatesh , U. P. Deshpande , M. Gangrade , V. Ganesan , Phys. B 2020, 590, 412145.

[23] L. Zeng , W. Zeng , Y. Jiang , X. Wei , W. Li , C. Yang , Y. Zhu , Y. Yu , Adv. Energy Mater. 2015, 5, 1401377.

[24] S. Lee , H. Lee , N. Ha , J. T. Lee , J. Jung , K. Eom , Adv. Funct. Mater. 2020, 30, 2000028.

[25] Y. Qi , Q. J. Li , Y. Wu , S. J. Bao , C. Li , Y. Chen , G. Wang , M. Xu , Nat. Commun. 2021, 12, 6347. 34732738 10.1038/s41467-021-26631-y PMC8566531

[26] N. B. Emerce , D. Eroglu , J. Electrochem. Soc. 2019, 166, A1490.

[27] X. Zhou , P. Gao , S. Sun , D. Bao , Y. Wang , X. Li , T. Wu , Y. Chen , P. Yang , Chem. Mater. 2015, 27, 6730.

[28] Q. Li , H. Liu , Z. Yao , J. Cheng , T. Li , Y. Li , C. Wolverton , J. Wu , V. P. Dravid , ACS Nano 2016, 10, 8788. 27564846 10.1021/acsnano.6b04519

[29] Q. Cai , Y. Li , L. wang , Q. Li , J. Xu , B. Gao , X. Zhang , K. Huo , P. K. Chu , Nano Energy 2017, 32, 1.

[30] F. Chen , C. Guo , H. Zhou , M. W. Shahzad , T. X. Liu , S. Oleksandr , J. Sun , S. Dai , B. B. Xu , Small 2022, 18, 2106352. 10.1002/smll.202106352 35060295

[31] R. Mukkabla , S. Deshagani , P. Meduri , M. Deepa , P. Ghosal , ACS Energy Lett. 2017, 2, 1288.

[32] Z. Shen , W. Zhang , S. Mao , S. Li , X. Wang , Y. Lu , ACS Energy Lett. 2021, 6, 2673.

[33] X. Zhao , L. Jiang , C. Ma , L. Cheng , C. Wang , G. Chen , H. Yue , D. Zhang , J. Power Sources 2021, 490, 229534.

[34] Y. Lei , X. Liang , L. Yang , J. Chen , L. Qu , K. Xu , J. Feng , Carbon 2022, 191, 122.

[35] J. Yang , H. Gao , D. Ma , J. Zou , Z. Lin , X. Kang , S. Chen , Electrochim. Acta 2018, 264, 341.

