# Supplementary material for: A Ternary Composite with Medium Adsorption Confirms Good Reversibility of Li‐Se Batteries
Source: Adv Sci (Weinh). 2023 Apr 14;10(16):2206962. doi: 10.1002/advs.202206962 (PMC10238200; doi:10.1002/advs.202206962)
Supplement: Supplementary file 1 — Supporting Information [file ADVS-10-2206962-s001.pdf]

ADVANCED SCIENCE
Open Access

Supporting Information

for *Adv. Sci.*, DOI 10.1002/advs.202206962

A Ternary Composite with Medium Adsorption Confirms Good Reversibility of Li-Se Batteries

Yi Li, Zhao Li, Liang Yue, Yi Zhang, Shuang Liu, Yubin Niu, Sam Zhang and Maowen Xu**

Supporting Information

A ternary composite with medium adsorption confirms good reversibility of Li-Se batteries

Yi Li, Zhao Li, Liang Yue, Yi Zhang, Shuang Liu, Yubin Niu, Sam Zhang, Maowen Xu*.*

Experimental Section

1. Preparation of $aTiO_2$@C, $aTiO_2/rTiO_2$/TiN@C, NC, TiN@C host materials

Typically, the MIL-125 nanomaterials were synthesized according to previous report.[1] Then, the as-prepared MIL-125 powder was put into a tubular furnace, after been pyrolyzed at a heating rate of 5 ^{o}C min^{-1} up to 600 ^{o}C for 3 h under Ar atmosphere, the powder was collected and soaked by 5 wt% HF solution to remove redundant TiO_2 species. After being centrifugated for 6 times by deionized water, the products were dried at 60 ^{o}C in an oven over night and the $aTiO_2$@C was harvested. When the powder was soaked by HF solution for a longer time, TiO_2 could be completely removed. Next, the $aTiO_2$@C was transferred into tubular furnace again. After undergoing a heating procedure (30°C to 800 °C at a heating rate of 5 °C min^{-1} at Ar atmosphere; maintained at 800 °C for 2h under NH_3 atmosphere), the ternary $aTiO_2/rTiO_2$/TiN@C was obtained. The ratio of $rTiO_2$ in the composite could be increased by lengthening the maintain time at 800°C in Ar gas, and the ratio of TiN could be increased by prolonging heating time at 800°C in NH_3 gas. The TiN@C host was synthesized by same heating procedure except for a higher end temperature at 900°C and longer maintaining time for 3h in NH_3. The N-doped porous carbon (NC) was prepared by azotizing above powder that has been completely removed TiO_2 through same heating procedure.

2. Method to accommodate Se

The Se powder is well mixed together with the above host materials respectively with weight ratio of 2:1, then the mixtures were put into a tube furnace keeping at 260 ^{o}C for 12 h at an inert atmosphere. After cooling to room temperature, the $aTiO_2$@C/Se, $aTiO_2/rTiO_2$/TiN@C/Se, NC/Se, TiN@C/Se were gained.

3. Adsorption experiment

Firstly, 20 mg Se and 10 mg Li_2Se were dissolved in 2 mL diethyl carbonate (DEC) solution, after continuous stirring and heating for about 3h until solution turned brown, the supernate was obtained as lithium polyselenides (Li_2Se_n) solution for further use. The visible adsorption experiment was conducted as follow. For the prior experiment, the commercial aTiO_2, rTiO_2, and TiN with a mass of 3 mg were put in an empty bottle respectively, and the diluted Li_2Se_n solution with same concentration was added in the bottles to observe the color. For the latter experiment, the as-prepared aTiO_2@C, NC, aTiO_2/rTiO_2/TiN@C, TiN@C, and acetylene black (AB) were taken for 5 mg and put in transparent glass bottles respectively. Then, 1.5 mL Li_2Se_n solution were taken and poured into 9 mL DEC to prepare a homogeneous dilute solution. The dilute Li_2Se_n solution were divided equally into the above glass bottles. Finally, the digital pictures and UV-vis absorption curves were taken after soaking.

4. Symmetrical battery measurement

The electrode slices were made through well grinding host materials (TiO_2@C, NC, aTiO_2/rTiO_2/TiN@C, TiN@C and AB respectively) and binder by a mass ratio of 9:1 in DI water, whereafter coating the mixture on aluminum foil pre-coated with carbon. Two identical electrode slices were employed as cathode and anode with same weight loading, and 20 μL of Li_2Se_n solution prepared above was dropped to the cathodic side and 15 μL electrolyte (1M $LiPF_6$ in a solution of EC/DEC with same volume) was dropped to the anodic side, meanwhile Celgard 2400 (PP) as separators. The Current-Voltage curves of the symmetric batteries were got between -1.5 to 1.5 V at a scan rate of 1 mV s^{-1}.

5. Materials Characterization

The morphology of materials was captured by a field emission scanning electron microscope (FESEM, JSM-7800F, Japan). A transmission electron microscope (TEM, JEM-2100, Japan) was used to acquire detailed morphology and lattice fringe image of materials. X-ray diffraction instrument (XRD, Bruker D8 Advance) was utilized to investigate crystalline structures of products and *in situ* electrochemical reaction. X-ray photoelectron spectroscopy (XPS) measurement was conducted by a spectrometer (Escalab 250xi, Thermo Scientific). A LabRAM HR Evolution (Horiba, Japan) was employed to acquire Raman spectrum with 532 nm laser excitation. The mass percentage of Se in the composites were studied by TGA (TGA-Q50) under

N_2 atmosphere, while $aTiO_2$, $rTiO_2$, and TiN in the host were test in air.

6. Electrochemical measurement

The active materials (TiO_2@C/Se, $aTiO_2$/$rTiO_2$/TiN@C/Se, NC/Se, TiN@C/Se), AB, and LA133 in a mass percentage of 70:20:10 were mixed with DI water as solvent to prepared a glossy slurry. The mixture was cast onto carbon-coated Al foil and then dried at 60 ^oC overnight to active as working electrode. Coin-type batteries (CR2032) were assembled by using Li metal as counter electrode, and a solution of 1M $LiPF_6$ in EC/DEC with same volume as electrolyte, and Celgard 2400 membrane as separators. The areal selenium loading for regular electrodes was about 0.6–1.0 mg cm^{-2} under the Electrolyte/Se (E/Se) ratio of 25 μL mg^{-1}. The specific capacities were calculated based on the mass of Se. Circulation and rate performances were conducted on the Neware and Land testing instrument within potential window of 1.0 V ~ 3.0 V. Cyclic voltammograms (CV) tests were obtained with CHI660E Electrochemical Workstation (Shanghai Chenhua Instruments, China).

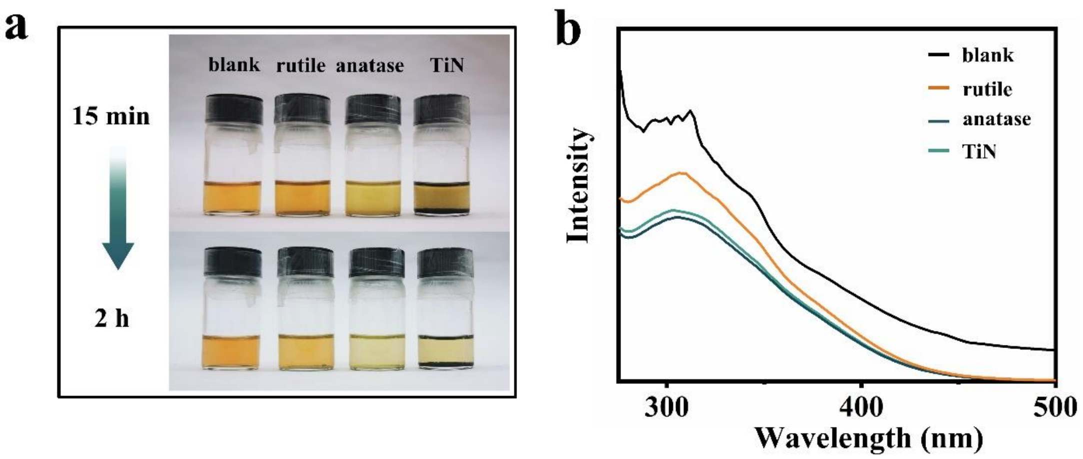

Figure S1. (a) Visualized adsorption tests of commercial $aTiO_2$, $rTiO_2$, and TiN, (b) UV-Vis tests for the upper solution after adsorbed.

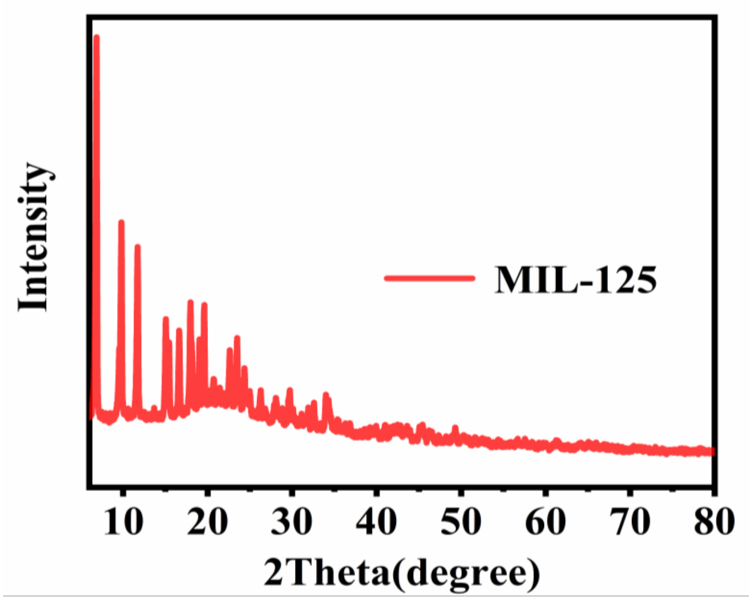

Figure S2. XRD pattern of as-prepared Ti-MOF.

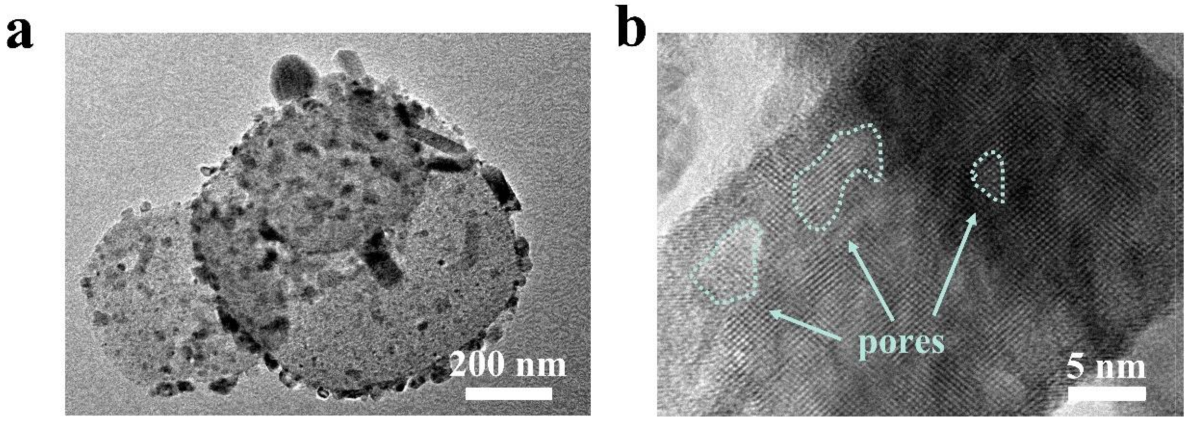

Figure S3. (a)(b) TEM images of aTiO_2/rTiO_2/TiN@C

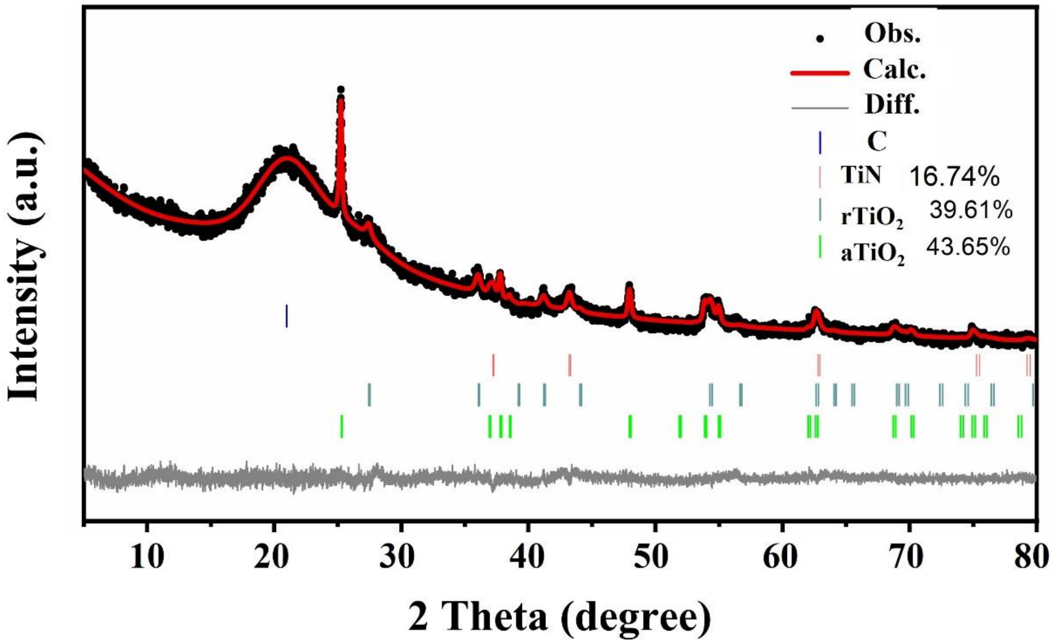

Figure S4. The XRD refinement curves of $aTiO_2/rTiO_2/TiN@C$.

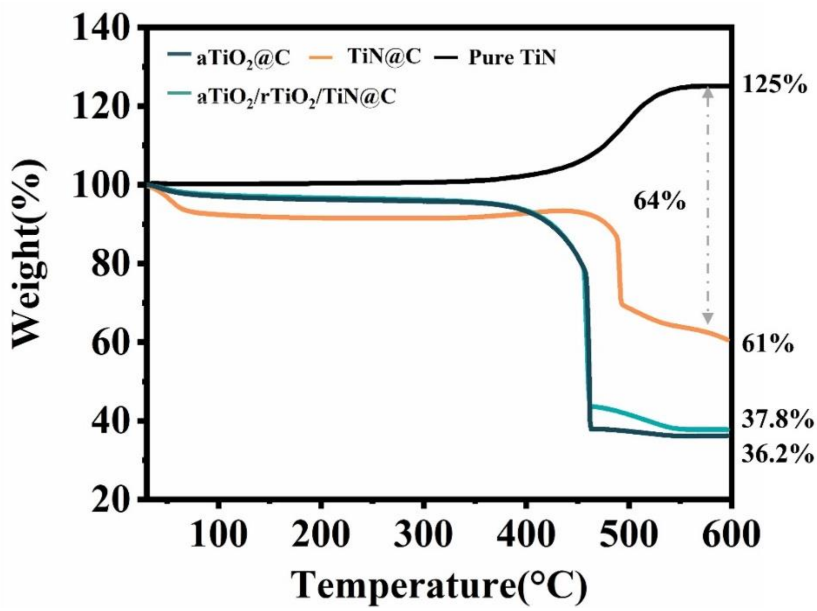

Figure S5. TGA curves of $aTiO_2/rTiO_2/TiN@C$, TiN@C, $aTiO_2@C$ and pure TiN at O_2 atmosphere.

The similar carbon content was made to reduce disturbance. The content of carbon, TiO_2, and TiN in host materials was characterized by TGA. For $aTiO_2@C$, the content of carbon is 63.8% and $aTiO_2$ is 36.2%. Pure TiN was taken to do TGA test, the result showed an increased mass of 125%. Then for TiN@C, the content of carbon should be 64% and TiN should be 36%. As calculated by FigS3, the mass ratio of $aTiO_2$, $rTiO_2$, and TiN in $aTiO_2/rTiO_2/TiN@C$ should be 43.7: 39.6: 16.7. Thus, the $aTiO_2$,

$rTiO_2$ could be deemed as 2.62 and 2.37 times of TiN respectively. The variation of weight could be loss of carbon with 0.25 times of TiN. Finally, the mass content of carbon and $aTiO_2$/ $rTiO_2$/TiN in $aTiO_2/rTiO_2$/TiN@C should be 63.9%, 36.1%.

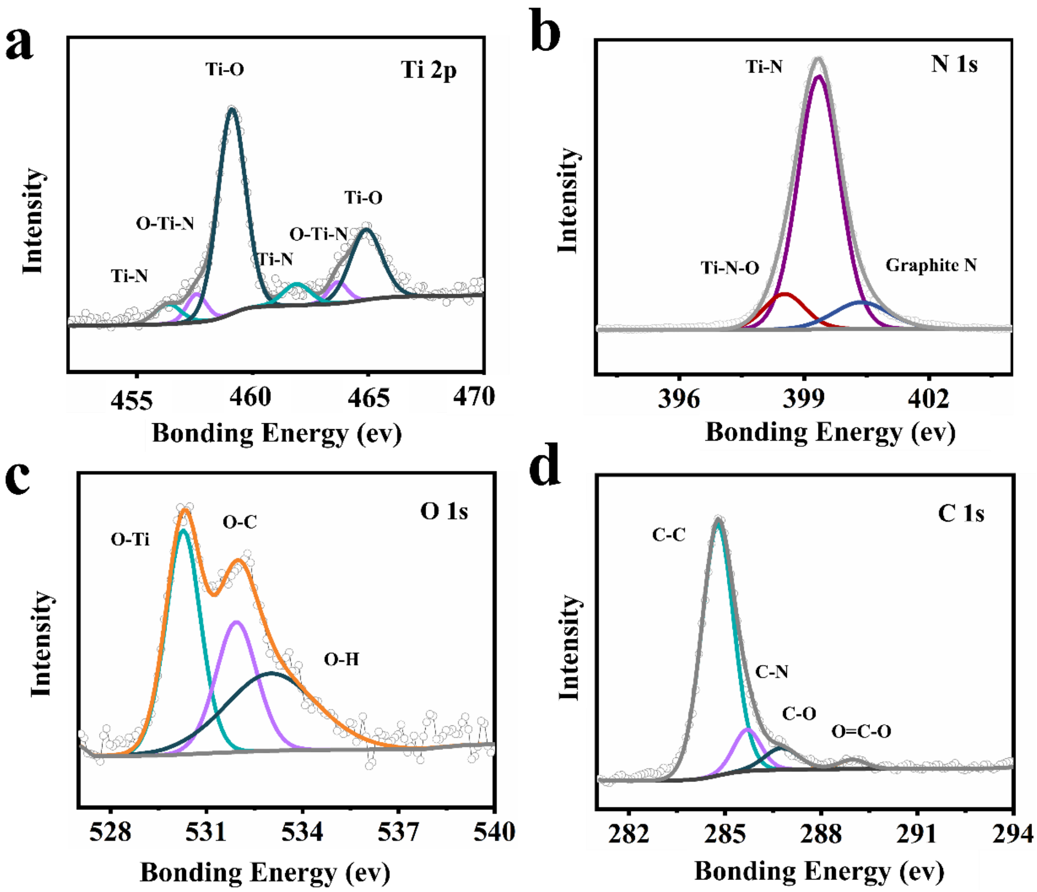

Figure S6. XPS spectra of $aTiO_2/rTiO_2$/TiN@C, (a) Ti 2p, (b) N 1s, (c) O 1s, (d) C 1s.

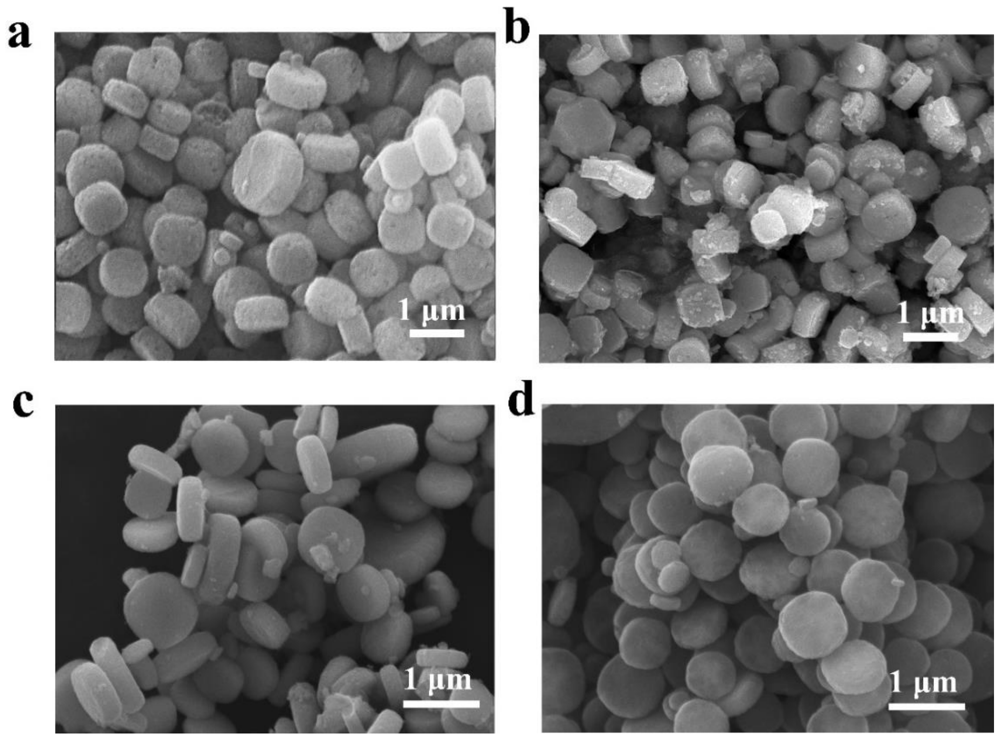

Figure S7. SEM images of (a) $aTiO_2$@C/Se, (b) $aTiO_2/rTiO_2$/TiN@C/Se, (c) TiN@C/Se, and (d) NC/Se.

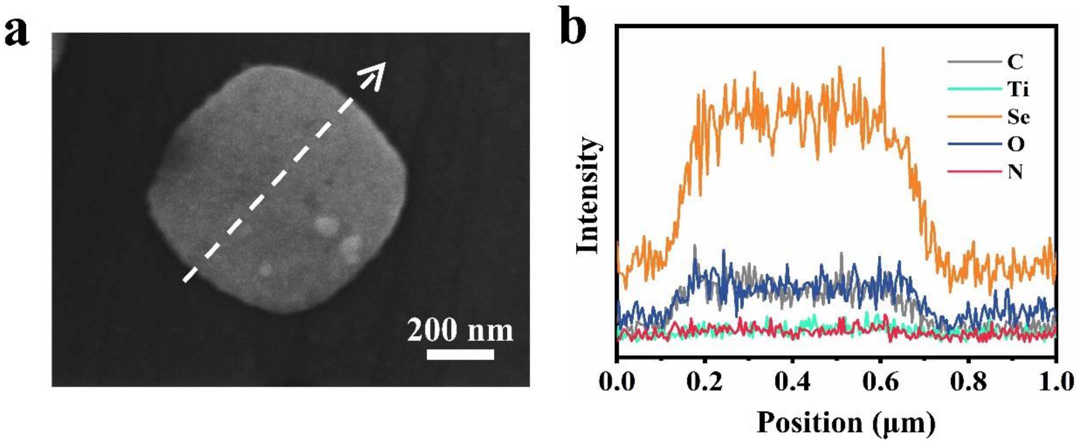

Figure S8. (a) SEM images, (b) liner EDS scanning of $aTiO_2/rTiO_2$/TiN@C/Se.

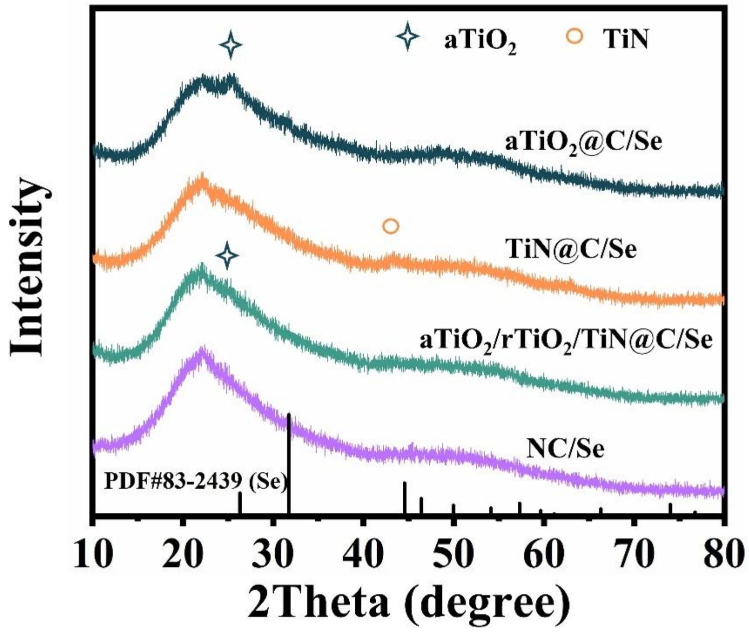

Figure S9. XRD curves of $aTiO_2$@C/Se, $aTiO_2/rTiO_2$/TiN@C/Se, TiN@C/Se, and NC/Se.

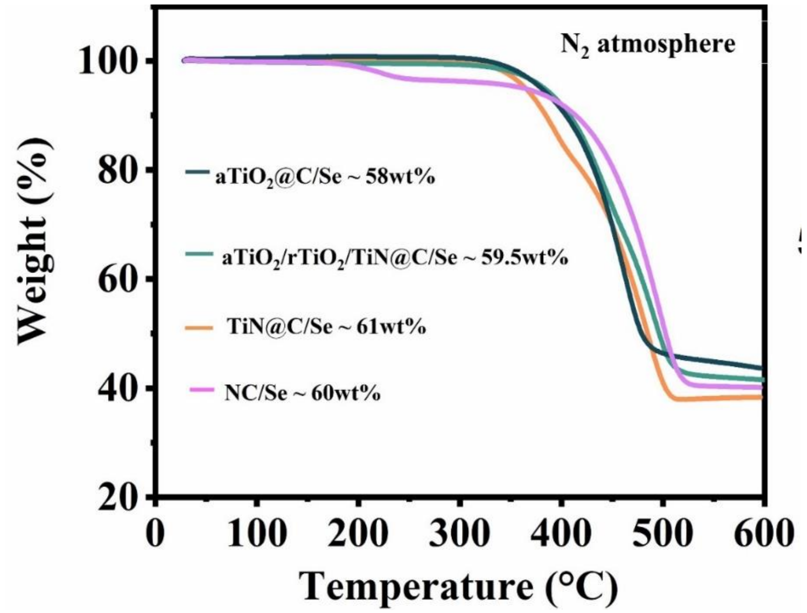

Figure S10. TGA curves of $aTiO_2$@C/Se, $aTiO_2/rTiO_2$/TiN@C/Se, TiN@C/Se, and NC/Se at N_2 atmosphere.

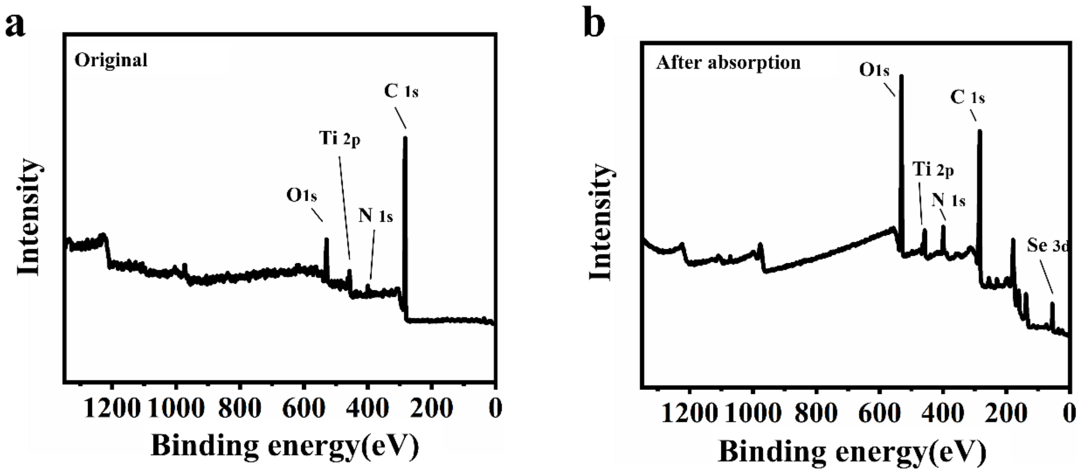

Figure S11. XPS survey of $aTiO_2/rTiO_2/TiN@C$ (a) original state (b) after absorption.

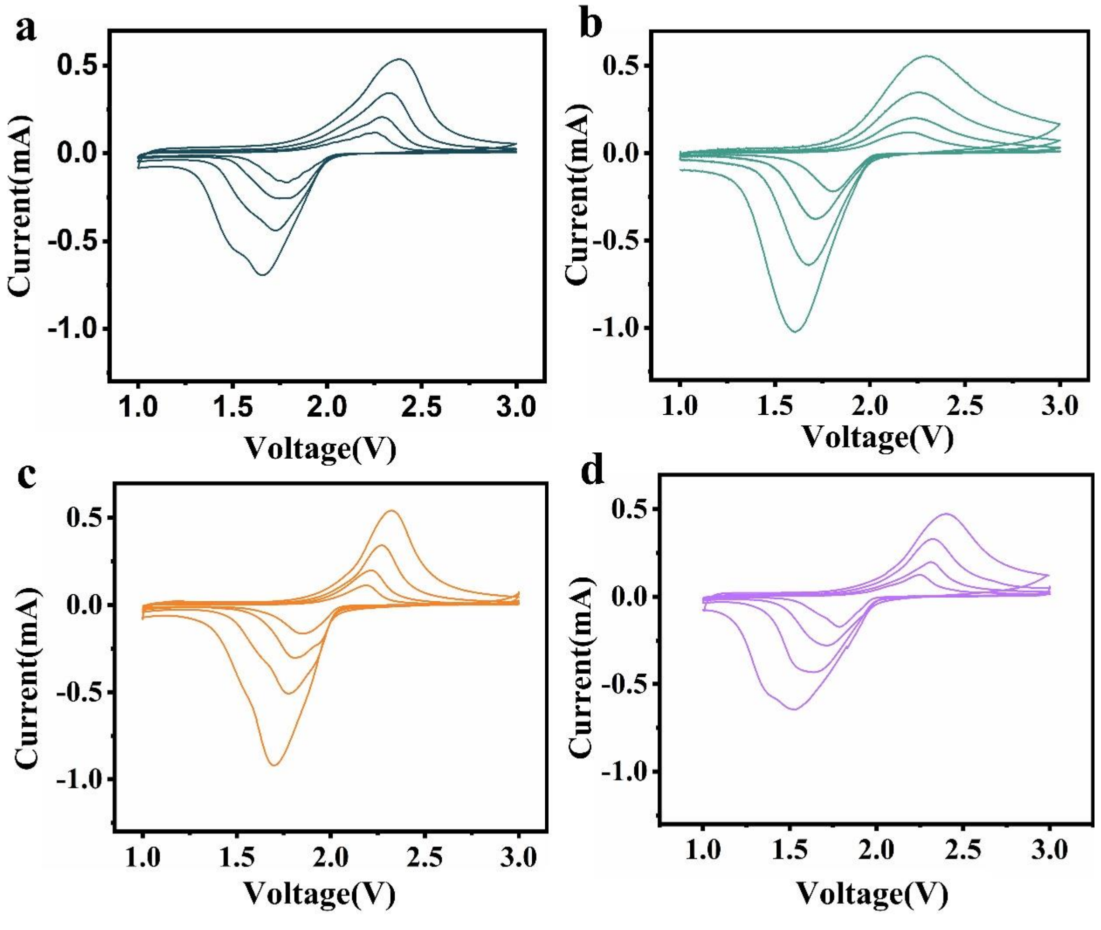

Figure S12. CV curves of (a) $aTiO_2@C/Se$, (b) $aTiO_2/rTiO_2/TiN@C/Se$, (c)TiN@C/Se, and (d) NC/Se electrodes at scan rate of 0.1 mV s^{-1}, 0.2 mV s^{-1}, 0.4 mV s^{-1}, and 0.8 mV s^{-1}.

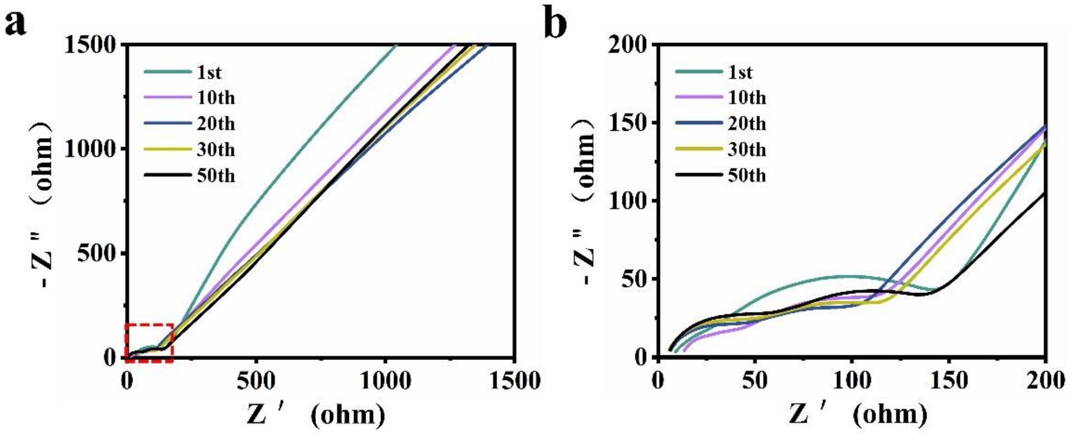

Figure S13. EIS results of (a) as-assembled $aTiO_2/rTiO_2/TiN@C/Se$ ‖Li batteries during cycling，(b) enlarged section in (a).

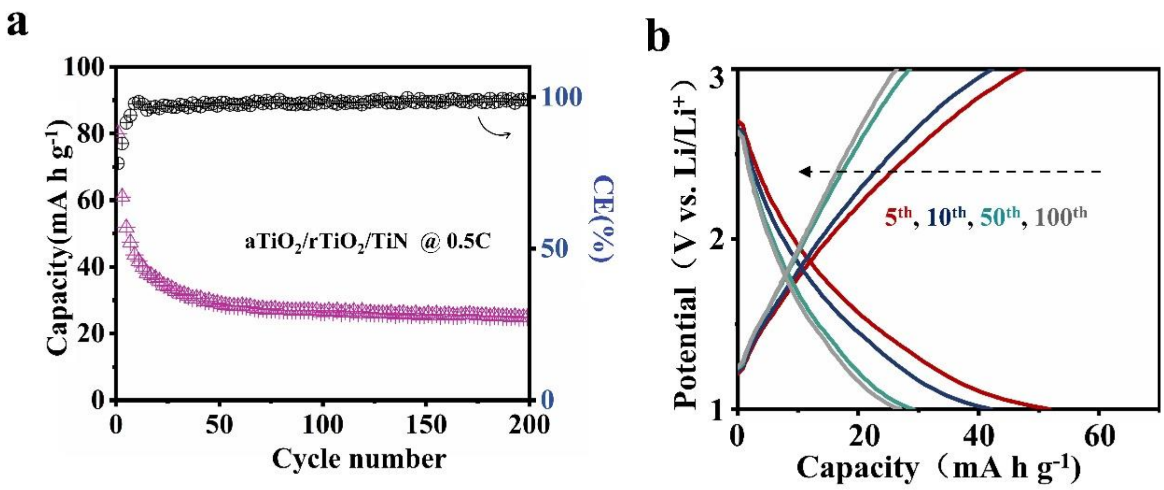

Figure S14. (a) Cycle performance and (b) charge-discharge profiles of bare $aTiO_2/rTiO_2/TiN@C$ electrode.

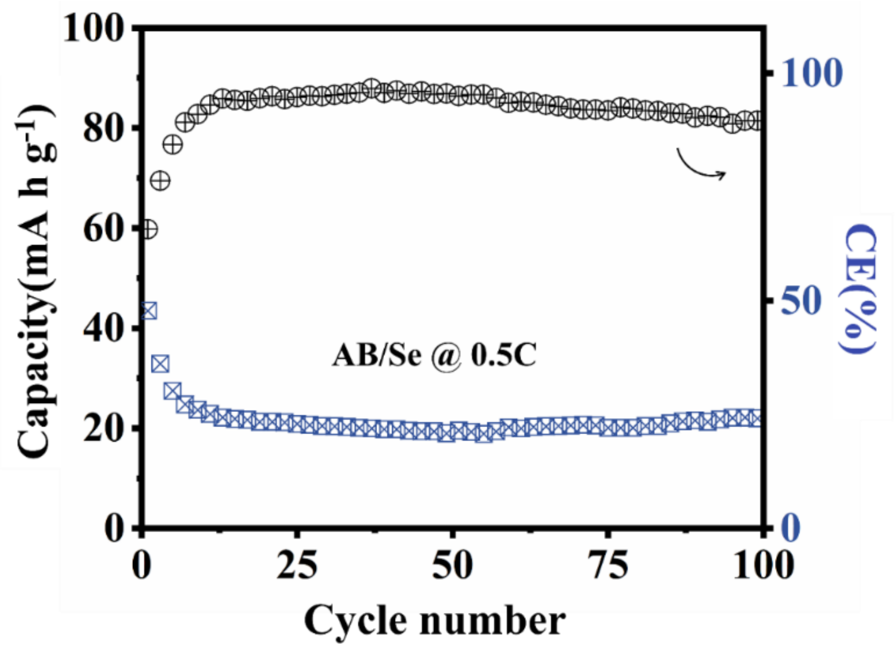

Figure S15. Cycle performance of AB/Se electrode.

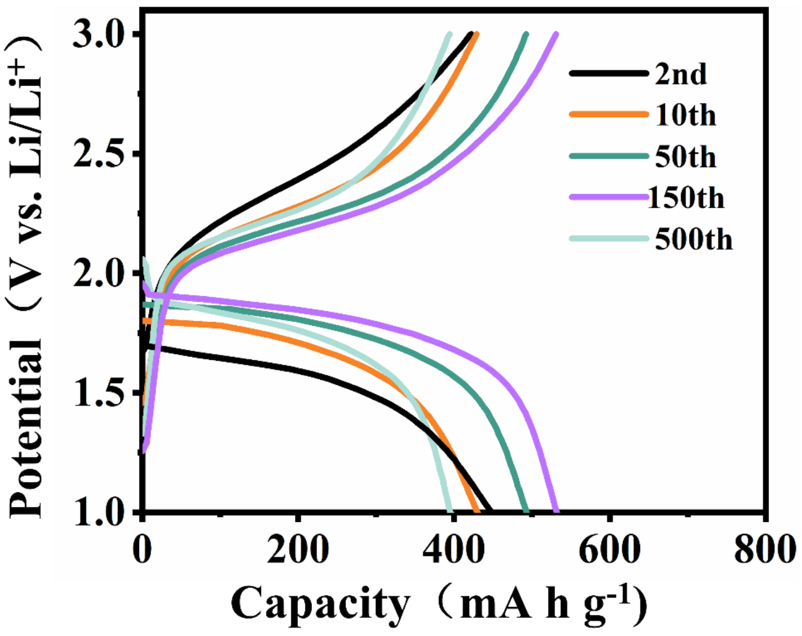

Figure S16. Charge-discharge profiles of $aTiO_2/rTiO_2/TiN@C/Se$ electrode at 2C.

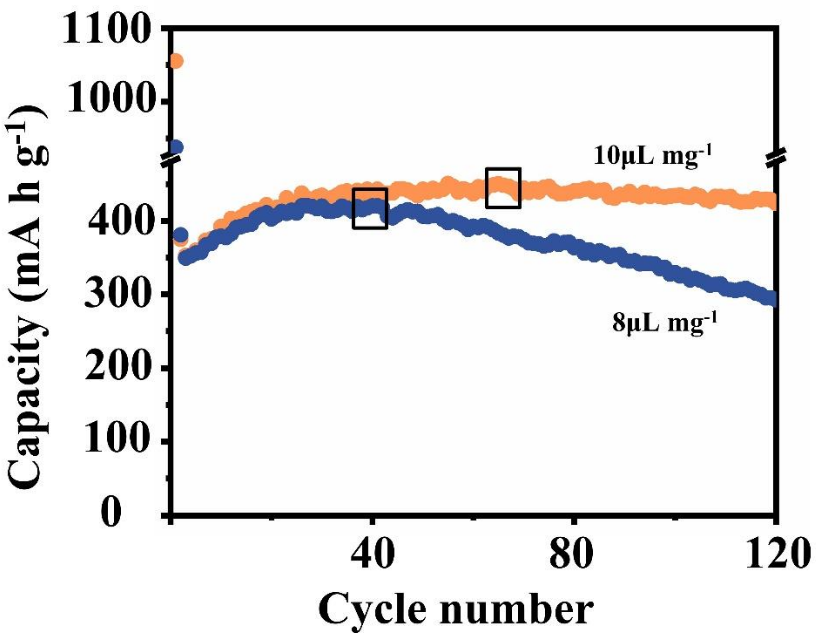

Figure S17. Cycling performance of $aTiO_2/rTiO_2/TiN@C/Se$ electrode on lean-electrolyte condition.

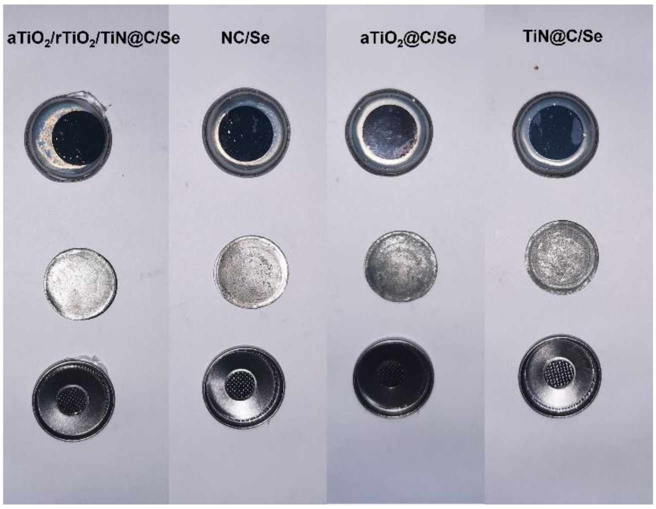

Figure S18. The optical photograph of disassembled cells cycled for 20 times.

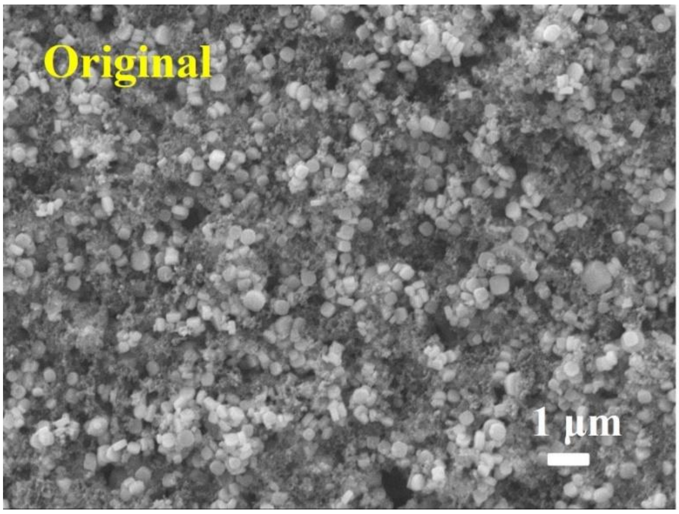

Figure S19. SEM image of original $aTiO_2/rTiO_2/TiN@C/Se$ electrode.

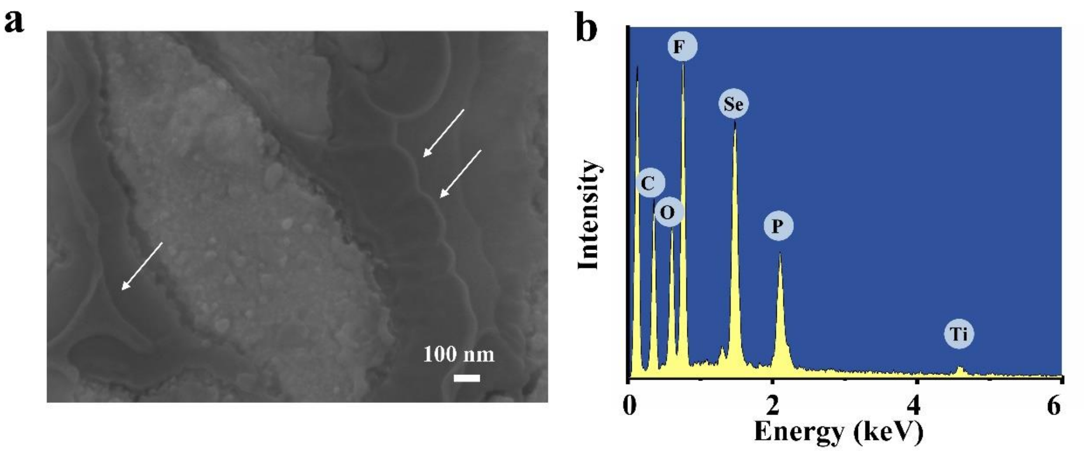

Figure S20. (a) High-resolution SEM image and (b) elemental composition of $aTiO_2/rTiO_2/TiN@C/Se$ electrode after cycled.

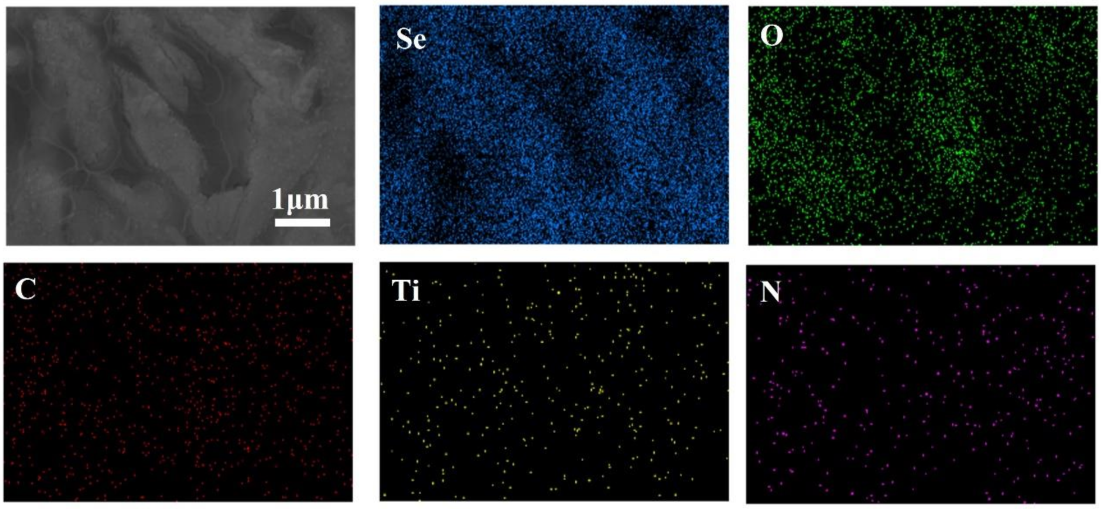

Figure S21. EDS element mapping of $aTiO_2/rTiO_2/TiN@C/Se$ electrode after cycled.

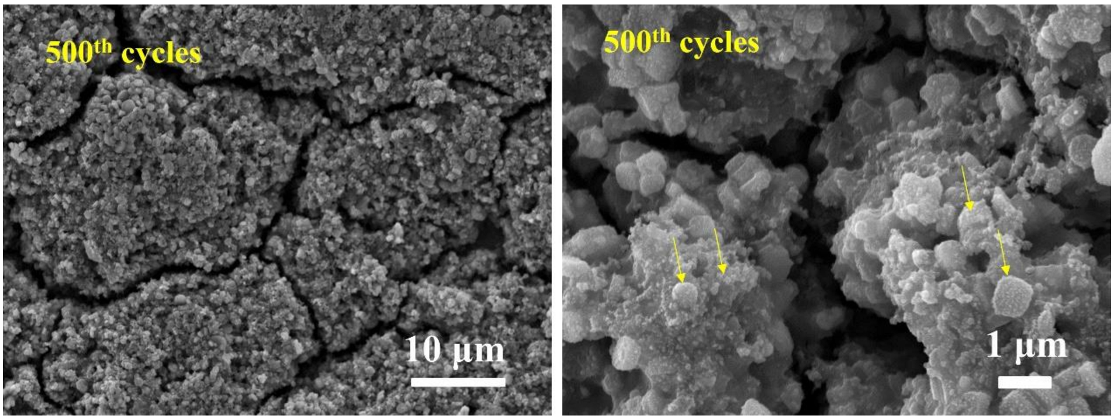

Figure S22. SEM images of $aTiO_2/rTiO_2/TiN@C/Se$ electrode after cycled for 500 times.

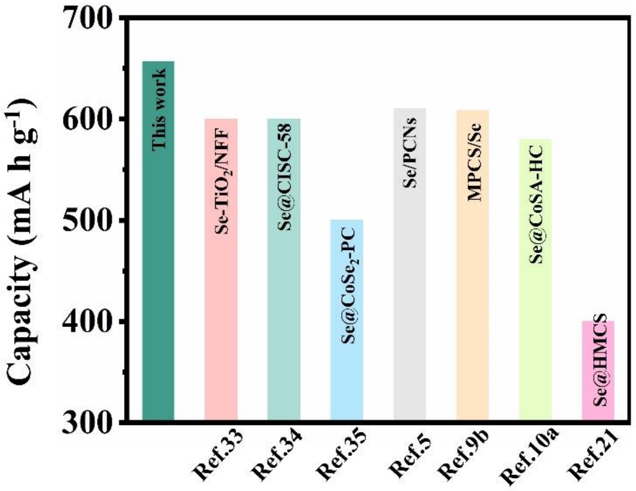

Figure S23 The comparison of electrochemical performance with other reported electrodes.

Reference

[1]Qi, C.; Xu, L.; Wang, J.; Li, H.; Zhao, C.; Wang, L.; Liu, T., Titanium-Containing Metal–Organic Framework Modified Separator for Advanced Lithium–Sulfur Batteries. *ACS Sustainable Chemistry & Engineering* **2020,** *8* (34), 12968-12975.
